# Prospective feasibility study of diagnostic CT-based simulation-free palliative radiotherapy

**DOI:** 10.1093/jrr/rrag056

**Published:** 2026-07-23

**Authors:** Takeshi Kashiwagi, Kiyonao Nakamura, Kazunori Tanaka, Tomohiro Kosuga, Kenta Fukumoto, Yuki Hanai, Shuji Ohtsu

**Affiliations:** Department of Radiation Oncology, Kyoto City Hospital, 1-2 Mibu Higashi Takada-cho, Nakagyo-ku, Kyoto 604-8845, Japan; Department of Radiation Oncology, Kyoto City Hospital, 1-2 Mibu Higashi Takada-cho, Nakagyo-ku, Kyoto 604-8845, Japan; Clinical Radiology Service Division, Kyoto City Hospital, 1-2 Mibu Higashi Takada-cho, Nakagyo-ku, Kyoto 604-8845, Japan; Clinical Radiology Service Division, Kyoto City Hospital, 1-2 Mibu Higashi Takada-cho, Nakagyo-ku, Kyoto 604-8845, Japan; Clinical Radiology Service Division, Kyoto City Hospital, 1-2 Mibu Higashi Takada-cho, Nakagyo-ku, Kyoto 604-8845, Japan; Clinical Radiology Service Division, Kyoto City Hospital, 1-2 Mibu Higashi Takada-cho, Nakagyo-ku, Kyoto 604-8845, Japan; Department of Radiation Oncology, Kyoto City Hospital, 1-2 Mibu Higashi Takada-cho, Nakagyo-ku, Kyoto 604-8845, Japan

**Keywords:** simulation-free radiotherapy, diagnostic CT, palliative radiotherapy, bone metastases, workflow efficiency

## Abstract

Conventional radiotherapy (RT) workflows require planning computed tomography (pCT) and treatment planning. However, these processes often take several days, leading to delays in RT initiation. Simulation-free radiotherapy (SFRT), which uses diagnostic CT (dCT) instead of pCT for treatment planning, may reduce the time from consultation-to-treatment delivery. However, the number of prospective studies conducted in Japan remains limited. This study prospectively evaluated the feasibility of SFRT in Japan. A single-center, single-arm prospective study (UMIN000054721) was performed in June 2024. This study included patients with vertebral metastases who were eligible for a single 8-Gy fraction. A dCT acquired within the last 56 days, without an immobilization device, and with an identifiable lesion, was required. The primary endpoint was the RT completion rate. The secondary endpoints included time in center (TIC), time from entering the consultation room to radiation beam-off, pain response and adverse events (AEs) at 4 and 12 weeks. Cone-beam CT (CBCT) verification was mandatory for patient setup. Five patients with six vertebral sites were enrolled. The RT completion rate was 100%. The mean TIC was 45 (range: 34–54) min. At 4 weeks, 25% of the patients presented with complete and partial pain responses. At 12 weeks, the complete response rate was 33%. None of the patients developed acute AEs. With appropriate patient selection and CBCT-guided verification, SFRT was feasible and safely implemented at a Japanese community hospital.

Clinical trial registration: UMIN-CTR: UMIN000054721

## INTRODUCTION

Palliative radiotherapy (RT) accounts for approximately half of all radiation treatments and plays an important role in achieving pain relief and local symptom control in patients with advanced cancer [1]. Bone metastases are a major cause of pain, impaired mobility and decline in performing activities of daily living, for which RT is widely used to achieve pain palliation and improve quality of life [2]. Bone metastases are among the most common indications of palliative RT. In particular, spinal metastases require the rapid delivery of RT because they carry additional risks of pain progression (PP), pathological fracture and neurological compromise. Conventional RT workflows require planning computed tomography (pCT) scan, body marking and subsequent treatment preparation. However, these processes often take several days, leading to delays in RT initiation [3].

Simulation-free radiotherapy (SFRT), which uses diagnostic CT (dCT) instead of pCT for treatment preparation, can reduce the time from consultation-to-treatment delivery while maintaining the quality of care. International clinical reports on SFRT have been increasing [4–7].

However, to the best of our knowledge, no prospective clinical reports on SFRT have been published in Japan, and the feasibility of SFRT in Japanese clinical practice remains unclear. The current study aimed to prospectively evaluate the feasibility of implementing dCT-based SFRT in a Japanese community hospital. To ensure the dosimetric validity of using dCT data for treatment planning, the Hounsfield unit (HU) values corresponding to known electron densities (ED) were compared across all five CT scanners at our institution prior to clinical implementation. In addition, this study also discussed the practical challenges associated with implementing SFRT in the Japanese clinical setting.

## MATERIALS AND METHODS

### Study design and patients

A single-center, single-arm prospective interventional study (UMIN-CTR: UMIN000054721) was conducted at our institution on 20 June 2024, after it was approved by the Institutional Review Board (approval no. 828) on the same day. The planned sample size was five patients. All patients provided a written informed consent prior to participation, and the study was conducted according to the principles of the Declaration of Helsinki.

### Eligibility criteria

The current study was limited to vertebral metastases and did not include nonvertebral nor nonbone metastases because vertebral metastases often allow therapy to be delivered with simple radiation beam arrangements. Patients who had a histologically confirmed malignancy and provided a written informed consent for palliative RT for vertebral bone metastases were eligible. The additional inclusion criteria were patients aged ≥18 years and those who could lie still in the supine position. A dCT acquired at our institution within the last 56 days, without a corset or other external device, and with the target lesion clearly identifiable, was required. Patients also needed to be eligible to receive a single 8-Gy fraction.

Patients with a previous history of RT to the treatment site or with a contraindication to RT, those who were pregnant, and those with severe psychiatric disorders were also excluded from the study.

### Comparison between CT scanners

Prior to the start of the current clinical trial, we evaluated five CT scanners (four CT scanners and one PET-CT scanner) at our institution, excluding our pCT scanner, using an electron density phantom (Model 062M, Sun Nuclear Corporation, Melbourne, FL, USA). We measured HU values on the phantom—which has known ED ranging from low to high—using both the pCT scanner and the other CT scanners, and calculated the differences between them.

### Treatment procedure

#### Radiotherapy planning

Treatment planning was performed using Pinnacle version 9.10 (Philips, Amsterdam, the Netherlands).

The dCT images were imported into the planning system, and the planning target volume (PTV) was contoured. Contouring of the clinical target volume (CTV) was not mandatory. When CTV delineation was performed, the vertebral body containing the lesion was generally defined as the CTV. Alternatively, it was also permissible to create a PTV that included the target vertebral body and one adjacent vertebral body above and below it within the rectangular treatment field, without creating a CTV. The margins from the CTV to the PTV and from the PTV to the field edges were individually determined by the radiation oncologist, typically within 5–10 mm, considering lesion location, proximity to the spinal cord and patient anatomy.

The dose reference point was placed at the isocenter or at an appropriate point within the spinal canal. A single 8-Gy fraction was prescribed to achieve a D_90_ of ≥8 Gy for the PTV while limiting the maximum dose to ≤115% (9.2 Gy). Fractionated irradiation was not permitted. If the maximum dose exceeded 9.2 Gy, a high-dose volume (V_9.2_ Gy) of <2 cm^3^ was considered acceptable.

Patients were treated with anteroposterior–posteroanterior (AP/PA) parallel-opposed pairs or a direct PA field. Advanced RT techniques, such as intensity-modulated radiotherapy (IMRT), volumetric-modulated arc therapy (VMAT) and stereotactic body radiotherapy (SBRT), were not allowed.

### Workflow

Figure 1 shows the trial schema. Prior to the initial consultation, patient eligibility was reviewed using data from the electronic medical record. If the patient met all the eligibility criteria, the RT plan was created using dCT instead of pCT.

**Fig. 1:**
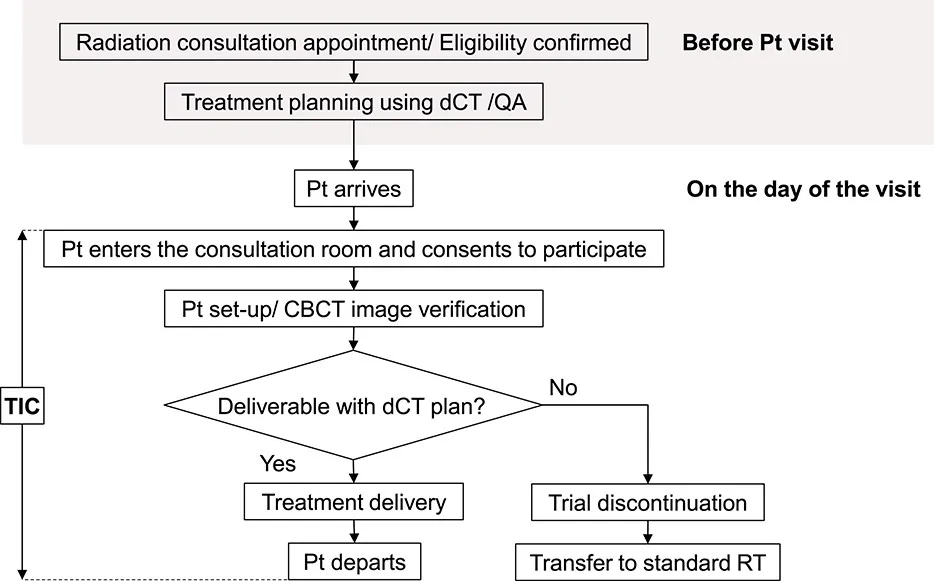
Workflow of SFRT using dCT for vertebral metastases. QA = quality assurance, Pt = patient, dCT = diagnostic computed tomography, TIC = time in center, CBCT = cone beam computed tomography.

On the day of the visit, the patient had a clinical consultation and provided consent to participate in the study. If the patients declined consent, they would receive standard palliative RT at our institution, including acquisition of pCT.

After obtaining informed consent and enrollment, patients were set up in the treatment room without body marking. Because the dCT couch differs from the treatment couch, a memory foam mattress was used during setup to reproduce the patient’s body contour and positioning as observed on the dCT (Fig. 2). Cone-beam CT (CBCT) image verification was mandatory for setup. The radiation oncologist and radiation therapist verified the setup together. SFRT was discontinued if CBCT images revealed that the target lesion no longer fit within the PTV due to deformation or enlargement of the target, or if there was a significant change in the body contour, and the standard RT workflow was followed. Final eligibility was determined at the discretion of the treating radiation oncologist, prioritizing treatment safety and dosimetric validity. After verification, treatment was delivered using the dCT plan.

**Fig. 2:**
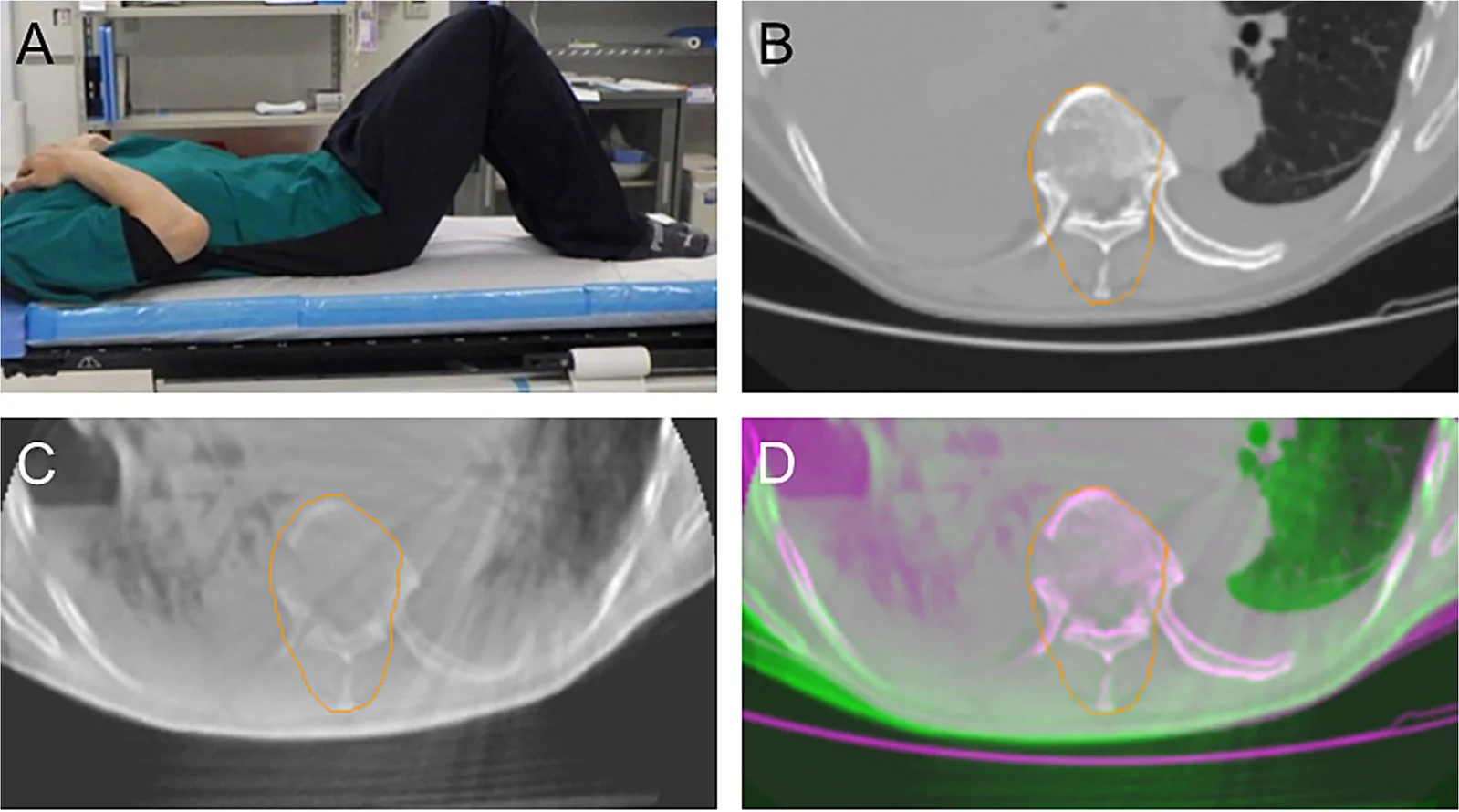
(A) Image showing a memory foam mattress on the treatment couch. (B) dCT image showing the body contour. (C) CBCT image showing the body contour during the treatment setup. (D) Fusion of dCT and CBCT showing reproducibility of the body contour. Pink indicates the dCT image, and green indicates the CBCT image. dCT = diagnostic computed tomography, CBCT = cone beam computed tomography.

### Endpoints and assessments

The primary endpoint was the RT completion rate.

The secondary endpoints included (i) time in center (TIC), defined as the time from entering the consultation room to radiation beam-off, (ii) pain response and (iii) adverse events (AEs), assessed at 4 and 12 weeks. Pain response was assessed at 4 and 12 weeks using the Numerical Rating Scale and the International Consensus Pain Response Endpoints [8]. The worst pain score within the last 3 days before each assessment, as self-reported by the patient, was used for analysis. AEs were graded according to the Common Terminology Criteria for Adverse Events version 5.0 [9].

### Statistical analysis

Owing to the feasibility design, the sample size was fixed at five patients. All statistical analyses were descriptive, and neither group comparisons nor hypothesis testing was conducted. Continuous variables were reported as means and medians (range), and categorical variables were expressed as counts and percentages.

## RESULT

### Comparison of HU values across CT scanners

Across all five dCT scanners, HU values corresponding to known ED closely matched the pCT reference values, except in the high-HU region. Low- to mid-density tissues (fat, water and muscle) showed a difference of 6.2 HU between dCT and pCT (−73.2 vs. −67 HU). In contrast, high-density tissues, such as the cortical bone, had a larger difference (49.6 HU) between dCT and pCT (1337.6 vs. 1288 HU), corresponding to ~3.9% of the reference values. This difference could have a minimal impact on dose calculation. Table 1 summarizes the comparison of HU values across CT scanners.

**Table 1:** Comparison of HU values across CT scanners

Tissue type	Electron density	pCT (HU)	dCT (HU), mean (range)	ΔHU
Fat	0.949	−67	−73.2 (−76 to −68)	−6.2
Water	1.00	−4	−3.2 (−21 to 5)	+0.8
Muscle	1.043	57	51.8 (50–55)	−5.2
Cortical bone	1.695	1288	1337.6 (1321–1370)	+49.6

pCT = planning CT, dCT = diagnostic CT, HU = Hounsfield unit.

### Characteristics of the patients

The target accrual was reached on 18 September 2024. During the study period, no patients declined SFRT after providing informed consent. The median follow-up period was 12 (range: 4–12) months. Table 2 shows the characteristics of the patients. Five patients with six vertebral metastatic sites were enrolled in this study. The median age was 72 (range: 47–83) years, and four were men. The primary tumors were lung cancer in three patients and renal cell carcinoma in two.

**Table 2:** Characteristics of the patients

Case	Age (years)	Sex	Primary tumor	Treated site	PS	dCT–RT interval (days)	TIC (min)
1	83	M	Lung	L4	1	13	43
2	59	M	RCC	Th2	1	10	34
3	82	F	RCC	Th11	2	44	48
4	72	M	Lung	Th1–3, Th8	2	6	54
5	47	M	Lung	Th1	2	1	44

PS = performance status, dCT = diagnostic CT, RT = radiotherapy, TIC = time in center, M = male, F = female, RCC = renal cell carcinoma, Th = thoracic, L = lumbar.

### Primary endpoint

Treatment planning was successfully completed in all five patients with six treatment sites. CBCT-based alignment revealed no setup errors requiring treatment plan modification. All plans were delivered without replanning or protocol deviations. Consequently, the RT completion rate was 100%. Table 3 presents the clinical outcomes. During the study period (20 June 2024 to 18 September 2024), RT was performed for 20 lesions in 16 patients with vertebral metastases and spinal dissemination that were considered potentially eligible for this workflow. Of these, 12 lesions in 9 patients received conventional RT, 2 lesions in 2 patients received SBRT, and 6 lesions in 5 patients received SFRT, with SFRT accounting for 30% of all cases.

**Table 3:** Clinical outcomes

Case	SFRT delivery	ICPRE Pain response		CTCAE grade	
		4 weeks	12 weeks	4 weeks	12 weeks
1	Yes	IR	IR	0	0
2	Yes	CR	CR	0	0
3	Yes	NE	NE	0	0
4	Yes	PR	IR	0	0
5	Yes	IR	NE	0	NE

SFRT = simulation-free radiotherapy, ICPRE = International Consensus Pain Response Endpoints, CTCAE = Common Terminology Criteria for Adverse Events, CR = complete response, PR = partial response, IR = indeterminate response, NE = not evaluable.

### Secondary endpoint

All patients completed the SFRT from the initial consultation to irradiation within a single day. The average TIC was 45 (range: 34–54) min. The average informed consent time was 21.75 (range: 17–27; unavailable for Study 3) min. In addition, retrospective review showed that the median interval from referral by the primary physician to consultation in the radiation oncology department was 8 (range: 1–28) days.

The evaluation of pain response excluded one patient who had no pain at baseline, and one patient died during follow-up. Hence, pain responses were evaluable in four patients at 4 weeks and in three patients at 12 weeks after treatment. At 4 weeks, the complete pain response (CR) rate was 25% (1/4), and the partial pain response (PR) rate was 25% (1/4). 25% (1/4) and 25% (1/4) of patients presented with PP and indeterminate responses (IR), respectively. At 12 weeks, the CR rate was 33% (1/3), and 67% (2/3) of the patients exhibited IR. None of the patients had PR or PP.

No acute or late AEs were observed during the follow-up period. Although the planned observation period was 12 weeks, three patients were followed-up for up to 56 (range: 17–56) weeks as part of routine clinical care. Pain responses and toxicity profiles are summarized in Table 3.

## DISCUSSION

This prospective feasibility study showed that SFRT could be safely implemented in a Japanese community hospital, with all patients completing treatment without acute toxicities. Moreover, the entire workflow—from the initial consultation to irradiation—was successfully completed within a single day. Based on these findings, SFRT may be a practical and time-efficient alternative to conventional simulation-based workflows in routine palliative RT. To the best of our knowledge, this study provides the first prospective evidence supporting the clinical implementation of SFRT in Japan.

The current study evaluated HU values corresponding to known ED across all five dCT scanners and the pCT at our institution. Results showed that the curves differed by only ~3.9% even in high-HU regions and high-density bones, such as the cortical bone, resulting in minimal impact on dose calculation. Previous studies have shown that retrospective recalculations comparing dCT and pCT-based plans (evaluated using PTV V95%, CTV V95% and V50%) revealed no clinically unacceptable dosimetric differences [10, 11]. Moreover, recalculations using the CBCT anatomy acquired during treatment showed that differences in PTV V95% generally ranged from −2% to 2.5% [4], indicating that an adequate target coverage can be achieved. Taken together, these findings support the dosimetric and geometric validity of SFRT. This study selected vertebral metastases as the treatment indication. Vertebral metastases provide geometrically and anatomically stable structures that ensure reproducible setup and allow treatment delivery with simple radiation beam arrangements. This selection is consistent with implementation guidance [12], which recommends anatomically stable sites such as the thoracic and lumbar spine for the initial SFRT implementation.

Previous reports from international institutions have described the real-world clinical implementation of SFRT. In the randomized DART trial [6], dCT-based radiation planning reduced the total time spent in the cancer center on the first day of treatment—from the first radiation appointment to completion of the first fraction—compared with conventional pCT-based workflows, without compromising plan deliverability or quality and without negative effects on the patient’s perception of acceptability. In addition, Schuler et al. reported their single-institution retrospective experience of 1000 SFRT cases, referred to as the SFRT-1000 cohort [7], in which clinically acceptable toxicity profiles were observed for palliative RT, including the IMRT and VMAT techniques. Moreover, Australian real-world reports have revealed that SFRT is feasible in resource-limited healthcare settings—particularly in rural and underserved areas [13]. These findings suggest that the SFRT workflow can be stably implemented in routine clinical practice.

The workflow in this study is characterized by its ability to shorten the time from consultation-to-treatment delivery. In fact, the concept of same-day consultation, planning and irradiation has existed internationally since the 1990s [14]. In recent years, rapid-access models, such as the FAST-METS trial, have implemented a rapid palliative workflow using dCT for pre-planning, with subsequent on-couch target and plan adaptation based on CBCT, thereby enabling same-day irradiation [15]. In this context, there has been an international movement that aimed to improve overall efficiency and reduce delays in palliative RT [16]. Our study contributes to this movement by providing prospective evidence from a real-world community setting in Japan.

In this study, same-day irradiation was feasible for all patients, with the TIC limited to ~45 min. Three workflow-related factors, supported by multidisciplinary collaboration, may have contributed to enabling same-day irradiation. First, the eligibility criteria were strictly applied, and only patients who could lie still in the supine position were included based on a review of data from the electronic medical record. For example, patients whose dCT images were acquired in the lateral position due to severe pain or those with dCT images whose quality was compromised due to motion artifacts were excluded. Second, radiation therapists coordinated irradiation schedules with medical consultation appointments, allowing patients to proceed directly to irradiation with minimal waiting time. Third, radiation oncology nursing staff reviewed the electronic medical records in advance to assess pain severity and the need for rescue medication. The patients who were anticipated to require rescue medication were contacted via telephone and instructed to bring their medications to ensure adequate pain control during irradiation. Furthermore, although same-day irradiation can be achieved within conventional pCT-based workflows, CT simulation and skin marking are typically required. In contrast, our SFRT approach eliminates both steps, thereby reducing the physical and operational burden of both patients and medical staff. Moreover, several clinical guidelines and real-world evidence support the use of single-fraction RT for painful bone metastases and metastatic spinal cord compression [17–19]. Our same-day SFRT workflow is consistent with these recommendations.

To promote SFRT in Japan, it is necessary to consider the unique characteristics of the Japanese healthcare delivery system. In rural areas, RT facilities are limited, and some patients are unable to access RT. Because SFRT reduces the number of required patient visits and alleviates the workload of medical staff, it has the potential to increase access to RT. In contrast, urban areas generally have a relatively large number of RT facilities, and many patients already receive RT. Therefore, unlike in rural areas, we do not expect the introduction of SFRT to increase the number of patients undergoing RT. However, because SFRT can reduce patient waiting times and improve treatment efficiency, its implementation may still offer certain advantages. Furthermore, regional hospitals typically have fewer staff members, resulting in simple SFRT workflows and relatively straightforward information sharing across departments. In contrast, large hospitals often involve many staff members in RT, making workflows more complex and potentially delaying information sharing, which may hinder the implementation of SFRT. Regardless of hospital size, SFRT requires workflows that differ substantially from those used in conventional RT based on pCT, and the optimal workflow is likely to vary across institutions. We believe that developing facility-specific workflows and reviewing them regularly will facilitate smoother implementation of SFRT.

The current study has four main limitations. First, this study had a small sample size and a short follow-up period. Consequently, long-term clinical outcomes—such as late pain relief and reirradiation rates—could not be adequately evaluated. Second, the irradiation technique was restricted to conventional AP/PA parallel-opposed fields. More recently, SFRT workflows incorporating advanced techniques such as IMRT/VMAT and on-couch adaptive RT have been reported, and these workflows have also been explored in the SBRT setting [11, 15, 20]. This study was an early-phase investigation into the feasibility of implementing this approach in real-world clinical practice in Japan for palliative spinal irradiation. In addition, the expansion to more advanced irradiation techniques remains an area for future research. Third, this was a single-institution study. The successful implementation may depend on institution-specific equipment and staffing. Therefore, the findings should be interpreted with caution when considering their generalizability. In this study, SFRT was performed using dCT images acquired within the same institution; thus, it remains unclear whether SFRT can be implemented using imaging data obtained from external institutions. Given the characteristics of the treatment planning system, we consider the following to be essential requirements for the safe and reliable implementation of SFRT using CT data from other institutions: (i) axial CT images without reconstruction, (ii) a field of view (FOV) encompassing the entire beam path and (iii) continuous DICOM image data with a slice thickness of ≤5 mm to ensure the accuracy of image-guided radiotherapy. Although the use of the same CT vendor or image acquisition under the same tube voltage may facilitate implementation without major technical difficulties, we do not consider these to be mandatory requirements. Fourth, although TIC was evaluated using the time from entering the consultation room to radiation beam-off, a detailed breakdown of TIC, such as setup time and room in/out time, was not prospectively recorded. Therefore, a detailed phase-by-phase analysis was not performed. Future studies including more detailed workflow data may help identify factors contributing to workflow efficiency in SFRT.

In conclusion, by combining appropriate patient selection and CBCT-guided verification, SFRT was feasible and safely implemented at a Japanese community hospital. Notably, a same-day consultation-to-treatment workflow could be achieved in routine clinical practice. In the future, continued prospective observational implementation with increased case numbers will be required to validate the clinical value and potential indications of this workflow.

## PRESENTATION AT A CONFERENCE

This study was presented at the 84th Annual Meeting of the Japan Radiological Society and the ESTRO 2025 Congress.
